# A Genetic Screen for Mutants with Supersized Lipid Droplets in *Caenorhabditis elegans*

**DOI:** 10.1534/g3.116.030866

**Published:** 2016-06-01

**Authors:** Shiwei Li, Shibin Xu, Yanli Ma, Shuang Wu, Yu Feng, Qingpo Cui, Lifeng Chen, Shuang Zhou, Yuanyuan Kong, Xiaoyu Zhang, Jialei Yu, Mengdi Wu, Shaobing O. Zhang

**Affiliations:** Laboratory of Metabolic Genetics, College of Life Sciences, Capital Normal University, Beijing 100048, China

**Keywords:** *C. elegans*, lipid droplet, peroxisome, forward genetic screen, *drop*

## Abstract

To identify genes that regulate the dynamics of lipid droplet (LD) size, we have used the genetically tractable model organism *Caenorhabditis elegans*, whose wild-type LD population displays a steady state of size with an upper limit of 3 μm in diameter. From a saturated forward genetic screen of 6.7 × 10^5^ mutagenized haploid genomes, we isolated 118 mutants with supersized intestinal LDs often reaching 10 μm. These mutants define nine novel complementation groups, in addition to four known genes (*maoc-1*, *dhs-28*, *daf-22*, and *prx-10*). The nine groups are named *drop* (lipid droplet abnormal) and categorized into four classes. Class I mutants *drop-5* and *drop-9*, similar to *prx-10*, are up-regulated in ACS-22-DGAT-2-dependent LD growth, resistant to LD hydrolysis, and defective in peroxisome import. Class II mutants *drop-2*, *drop-3*, *drop-6*, and *drop-7* are up-regulated in LD growth, are resistant to LD hydrolysis, but are not defective in peroxisome import. Class III mutants *drop-1* and *drop-8* are neither up-regulated in LD growth nor resistant to LD hydrolysis, but seemingly up-regulated in LD fusion. Class IV mutant *drop-4* is cloned as *sams-1* and, different to the other three classes, is ACS-22-independent and hydrolysis-resistant. These four classes of supersized LD mutants should be valuable for mechanistic studies of LD cellular processes including growth, hydrolysis, and fusion.

The fat triacylglycerol (TAG) is stored in lipid droplets (LDs), a type of cellular organelle conserved across eukaryotes (Martin and Parton 2006). While the importance of LD in cellular fat storage and fat mobilization has emerged through several lines of research, mechanisms underlying basic LD cellular processes such as *de novo* formation, growth, fusion, and hydrolysis are still poorly understood (Ploegh 2007; Walther and Farese 2012; Yang *et al.* 2012). Changes in the processes of *de novo* formation, growth, fusion, or hydrolysis may lead to changes in the number or size of LDs. Thus, size change may serve as a convenient morphological entry point to dissect the underlying metabolic pathways that anabolize and catabolize fat, and to uncover the protein-lipid membrane machinery that channels fat into and out of LDs.

Candidate gene approaches based on gene knockouts or proteomics have led to the identification and an initial understanding of the roles of LD surface proteins such as PLINs, ATGL, DGAT, and Cidec in the regulation of LD hydrolysis, growth, and fusion (Walther and Farese 2012). As confirmed by several studies, functional perturbation of LD processes through gene manipulation often leads to changes in LD size, number, or distribution. Thus, genetic screens for morphological LD alterations may be used as a first step to discover genes and to relate gene functions to cognate LD processes. Along this line, whole genome reverse genetic screens have been undertaken to identify genes whose functional loss leads to changes in LD size, number, or spatial distribution at the single-cell level. For example, whole genome RNAi screens in *Drosophila* S2 cells uncovered the involvement of Arf1-COPI complex in LD morphology and a role of CTP:phosphocholine cytidylyltransferase (CCT) in typical LD fusion (Beller *et al.* 2008; Guo *et al.* 2008; Krahmer *et al.* 2011). Deletion mutant library screens in the single-celled yeast *Saccharomyces cerevisae* revealed the roles of SEIPIN and phosphatidic acid metabolism enzymes in typical LD fusion (Szymanski *et al.* 2007; Fei *et al.* 2008, 2011). Unbiased saturated forward genetic screens for morphological LD mutants in multicellular systems, however, have rarely been reported.

Through a combination of biochemical, histological, and microscopy approaches, Zhang and colleagues recently demonstrated that LDs rather than lysosome-related organelles (LROs) are the fat storage organelles in *Caenorhabditis elegans* (Zhang *et al.* 2010b). In *C. elegans*, vital Nile Red staining labels LROs but not LDs. However, postfix Nile Red staining and postfix Oil-Red-O staining specifically label LDs (Zhang *et al.* 2010a,b). These results also demonstrated that “fat stores accessible to postfix Nile Red” and “fat vesicles distinct from LROs” defined in two earlier reports (Brooks *et al.* 2009; O’Rourke *et al.* 2009) were actually LDs, and, explained why staining intensity of postfix Nile Red and postfix Oil-Red-O but not of vital Nile Red may be used as a semiquantitative measurement of relative TAG level (Schroeder *et al.* 2007; Brooks *et al.* 2009; O’Rourke *et al.* 2009). In contrast to vital Nile Red, vital BODIPY labels LDs in addition to LROs. For this reason, BODIPY fluorescence intensity may not be used to quantify relative TAG level. However, vital BODIPY is still qualitatively useful for labeling LDs in wild type and for labeling LDs specifically when LROs are abolished in a *glo-4* mutant background (Zhang *et al.* 2010a,b).

As the only qualitatively reliable vital LD dye currently available, BODIPY should be valuable for the fast identification of mutants with LD size changes in forward genetic screens of a large number of live animals. This argument is supported by the fact that in a screen of 3.6 × 10^4^ haploid genomes, vital BODIPY labeling allowed the isolation of 11 *bona fide* supersized LD mutants corresponding to four genes: *maoc-1*, *dhs-28*, *daf-22*, and *prx-10* (Butcher *et al.* 2009; Zhang *et al.* 2010a). MAOC-1, DHS-28, and DAF-22 are three consecutive enzymes for peroxisomal fatty acid β-oxidation. PRX-10 is responsible for the import of peroxisome matrix enzymes including MAOC-1, DHS-28, and DAF-22. Loss-of-function mutation of these proteins results in a decrease of fatty acid breakdown and an increase of fatty acid storage in the form of TAG. The increase of TAG storage is expressed as an enhancement of LD growth concurrent with an inhibition of LD hydrolysis. The net effect of growth and hydrolysis is accumulated across developmental stages, resulting in supersized LDs 3–10 μm in diameter by larval stage L4 and even larger in adults (Zhang *et al.* 2010a). The enhanced LD growth is dependent on the TAG-synthesis enzyme complex ACS-22/FATP1-DGAT-2, which is conserved between *C. elegans* and mammals (Xu *et al.* 2012). Whether the BODIPY-based forward genetic screen was saturated was not tested.

To fully exploit the BODIPY-based strategy for isolating supersized LD mutants, we have now conducted a saturated genetic screen with several innovations: (1) increasing the haploid genome number 18.6 times to 6.7 × 10^5^, (2) using both ethylmethanesulfonate (EMS) and ethylnitrosourea (ENU) as mutagens, (3) applying a novel temperature-sensitive mutant selection regimen, (4) using high-copy transgenic lines expressing wild-type MAOC-1/DHS-28/DAF-22 as a mutagenesis background in order to target only new genes, and (5) using *acs-22* mutant as a mutagenesis background to enrich mutants of novel genes that are ACS-22-independent. In this way, we isolated in total 118 mutants. Forty-five are new alleles of four previously reported genes for peroxisome function: *maoc-1*, *dhs-28*, *daf-22*, and *prx-10*; the other 73 define nine novel complementation groups that are named *drop* (lipid droplet abnormal) and are categorized into four classes. These four classes of mutants represent valuable models for the regulation of LD growth, hydrolysis, and fusion.

## Materials and Methods

### C. elegans strains and culture conditions

The wild-type strain was N2 Bristol. The mapping strain was a Hawaiian isolate CB4856. All strains were raised on nematode growth media (NGM) plates seeded with OP50 *Escherichia coli*. Growth temperature was 20° if not otherwise indicated. Mutant alleles and transgenic lines published before or obtained from other sources are: *maoc-1(hj3) II*, *daf-22(ok693) II*, *prx-10(hj21) III*, *glo-4(ok623) V*, *acs-22(tm3236) X*, *dhs-28(tm2581) X*, *hjIs37[vha-6p::mrfp::pts1]*, *hjIs73[vha-6p::gfp::daf-22]*, and *hjSi56[vha-6p*::*3xflag*::*tev*::*gfp*::*dgat-2] IV*.

The 13 complementation groups of 118 mutant alleles (reference alleles in bold) isolated in the current study are:

*maoc-1(ssd64*, *ssd65*, *ssd66*, *ssd67*, *ssd196) II*
*dhs-28(ssd39*, *ssd40*, *ssd41*, *ssd42*, *ssd43*, *ssd44*, *ssd45*, *ssd46*, *ssd47*, *ssd48*, *ssd50*, *ssd197*, *ssd207*, *ssd208*, *ssd209) X*
*daf-22(ssd51*, *ssd52*, *ssd53*, *ssd54*, *ssd55*, *ssd56*, *ssd57*, *ssd58*, *ssd59*, *ssd60*, *ssd61*, *ssd62*, *ssd63*, *ssd195*, *ssd198*, *ssd200*, *ssd201*, *ssd202*, *ssd204*, *ssd205*, *ssd210*, *ssd215*, *ssd217) II*
*prx-10(ssd68*, *ssd69) III*
*drop-1(ssd1*, ***ssd2***, *ssd3*, *ssd4*, *ssd5*, *ssd6*, *ssd7*, *ssd8*, ***ssd9***, *ssd10*, *ssd11*, *ssd12*, *ssd79*, *ssd80*, *ssd81*, *ssd82*, *ssd83*, *ssd84*, *ssd85*, *ssd86*, *ssd87*, *ssd88*, *ssd203*, *ssd212*, *ssd219*, *ssd220*, *ssd221*, *ssd222*, *ssd223*, *ssd224*, *ssd225*, *ssd226*, *ssd227*, *ssd228*, *ssd229*, *ssd230*, *ssd231*, *ssd255) II*
*drop-2(ssd13*, ***ssd14***, *ssd15*, *ssd16*, *ssd17*, *ssd18*, *ssd19*, *ssd20*, *ssd21*, *ssd22*, *ssd23*, *ssd24*, *ssd25*, *ssd26*, *ssd27*, *ssd28*, *ssd29*, *ssd30*, *ssd31*, *ssd32*, *ssd33*, *ssd211) IV*
*drop-3(ssd34*, *ssd35*, ***ssd36***, *ssd37*, *ssd74) IV*
*drop-4(****ssd206****) X*
*drop-5(****ssd72***, *ssd216) X*
*drop-6(ssd38*, ***ssd73****) X*
*drop-7(****ssd75****)*
*drop-8(****ssd89****) III*
*drop-9(****ssd213****) II*

Also generated in this study are *dgat-2(ssd90) V*, *ssdIs1[dhs-28; maoc-1; 3xflag::daf-22::SL2::mrfp::pts1] V*, *ssdIs5[dhs-28; maoc-1; 3xflag::daf-22::SL2::mrfp::pts1] X*. *ssdIs1* and *ssdIs5* were made by UV integration of an extrachromosomal array *ssdEx1[dhs-28; maoc-1; 3xflag::daf-22::SL2::mrfp::pts1]*, which was derived from an injection of plasmid *vha-6p::dhs-28*, plasmid *vha-6p::maoc-1*, and plasmid *vha-6p::3xflag::daf-22::SL2::mrfp::pts1*, each at 10 ng/μl.

### Mutagenesis and screening

N2 Bristol (WT), *ssdIs1/ssdIs5* (*Is[mddm]*), *acs-22(tm3236)* were used as mutagenesis backgrounds. Synchronized L4 hermaphrodites of each background were mutagenized with 50 mM EMS (ethylmethanesulfonate, #M0880-10G, Sigma-Aldrich) or 0.5 mM ENU (ethylnitrosourea, #N3385-1G, Sigma-Aldrich) for 4 hr. ENU was stored and applied essentially as described in De Stasio and Dorman (2001). Healthy mutagenized P0 animals were picked onto 6 cm NGM/OP50 plates, 3 to 5 P0s per plate. On each plate, P0s were allowed to lay 150–200 F1s. Each plate of F1s was an independent pool of haploid genomes. For each screen series, about 300 pools/plates were set up. F1s on each plate were allowed to lay enough F2 eggs. The plate was then washed, leaving only F2 eggs. F2 eggs were allowed to hatch for 2–4 hr to produce 1500–3000 synchronized stage L1 larvae. Synchronized F2 larvae were transferred onto a BODIPY-layered NGM/OP50 plate to grow for 2–3 d at 15° or 20°, and were then transferred to 30^o^ for 4 hr. F2s were screened under a Nikon SMZ1500 stereo microscope in green fluorescent protein (GFP) fluorescence and bright field channels. Candidate supersized LD mutants were picked out by the presence of enlarged BODIPY-positive globular structures in intestinal cells. From each pool only a single isolate was kept. Candidate isolates identified by BODIPY and bright field visualization were further tested by postfix Oil-Red-O staining, postfix Nile Red staining, and GFP::DGAT-2 marking.

### Vital BODIPY staining and visualization

A total of 100 μl of 5 μM green BODIPY (#D-3823, Invitrogen) or red BODIPY (#D-3835, Invitrogen) in 1X phosphate buffered saline (PBS, pH 7.2) was added to the ∼2 cm-in-diameter OP50 bacteria lawn of a 6 cm NGM plate. The plate was immediately dried in a laminar flow hood to grow and stain animals. For screening, freshly hatched L1s were loaded onto the plate to grow to L4 and adult stages. BODIPY fluorescence was examined under a Nikon SMZ1500 stereo microscope. For high power confocal imaging, young adult animals were loaded onto the BODIPY plate to produce progeny and late L4 stage progeny were then mounted in 0.2 mM levamisole on agarose-padded slides for imaging.

### Postfix Oil-Red-O staining and bright field imaging

Late L4 stage animals were fixed in 1% paraformaldehyde/PBS for 30 min with rocking. Samples were then immediately frozen on dry ice/ethanol and thawed with running tap water. Samples were frozen and thawed again, three times in total. Samples were washed three times with 1X PBS, dehydrated in 60% isopropanol for 2 min, stained with 0.5 ml 60% Oil-Red-O (#O0625-25G, Sigma-Aldrich) working solution for 30 min with rocking. Oil-Red-O stock solution was dissolved in isopropanol at a concentration of 0.5 g/100 ml and was equilibrated for several days. Oil-Red-O working solution was prepared fresh by mixing 60% volume of stock with 40% volume of water. The mix was equilibrated for 10 min, and then filtered with a 0.22 μm spin-filter. Stained samples were then washed three times with 1X PBS, rehydrated in 1X PBS, and mounted in 1X PBS onto agarose-padded slides for imaging. Some Oil-Red-O color images were acquired on a Zeiss Axiovert 200M compound microscope using a FLUAR 40X/1.30 Oil Iris objective. The camera was a RETIGA-SRV FAST1394 device (QIMAGING) controlled by Image-Pro Plus 7.0 software. Other Oil-Red-O images were acquired on a Zeiss Imager M1 compound microscope using an EC Plan-Neofluar 40X/1.30 Oil DIC objective. The camera used was a Zeiss AxioCam HRc5 controlled by Zeiss Axiovision LE 4.0 software.

### Postfix Nile Red staining

Late L4 animals were fixed, freeze-thawed, washed, and dehydrated in the same way as in postfix Oil-Red-O staining. Animals were then stained with 0.5 ml of 1 μg/ml Nile Red (#N1142, Invitrogen) in 60% isopropanol for 30 min. Stained samples were then washed three times with 1X PBS, rehydrated in 1X PBS, and mounted in 1X PBS onto agarose-padded slides for confocal imaging.

### Confocal fluorescence imaging and spectral analysis

All confocal fluorescence images were acquired on a Zeiss LSM780 inverted confocal microscope with a Plan-Apochromat 63X/1.40 Oil DIC M27 or EC Plan-Neofluar 40X/1.30 Oil DIC M27 objective. Channel mode imaging was applied to BODIPY, GFP::DGAT-2, GFP::DAF-22, and mRFP::PTS1 fluorescence. For GFP and green BODIPY, the excitation laser was 488 nm and emission was collected at 493–598 nm; for mRFP and red BODIPY, excitation was 543 nm, and emission 582–728 nm. Time-lapse channel mode imaging of GFP::DGAT-2 was at one frame every 30 sec. Each frame was a 9 μm Z-stack and was exported as an extended focus view. Lambda mode imaging and spectral analysis were conducted essentially in the same way as that described in Zhang *et al.* (2010b). For lambda mode imaging of GFP::DGAT-2 and autofluorescence, excitation was 488 nm and emission was 495–647 nm at 9 nm per channel. GFP and autofluorescence signals were separated using linear unmixing. For lambda mode imaging of postfix Nile Red fluorescence, excitation was 514 nm and emission was 540–691 nm at 9 nm per channel. LD fluorescence was separated from the diffuse background fluorescence using linear unmixing.

### Genetic background cleaning

All strains were crossed at least four times before phenotype analysis. Mutants isolated from N2 Bristol/WT background were backcrossed four times with N2. Mutants isolated from *acs-22(tm3236)* background were backcrossed four times with *acs-22(tm3236)* and then segregated out of *acs-22(tm3236)*. Mutants isolated from *Is[mddm]* were backcrossed four times with *Is[mddm]* and then segregated out of *Is[mddm]*.

### Complementation test

The 118 mutants were tested for complementation against *maoc-1(hj13)*, *dhs-28(tm2581)*, *prx-10(hj21)*, and *daf-22(ok693)*. Forty-five mutants were identified as new alleles of these four groups. The other 73 mutants were tested against each other and were sorted into nine groups.

### maoc-1/dhs-28/daf-22/prx-10 genomic DNA sequencing

The primers used for amplifying and sequencing the four genes’ genomic DNA are listed below. Exons, intron splice donor sites, and intron acceptor sites were covered for each gene.

*maoc-1*: 0062F: GGTTTTTGTTTCTTCTCCGAC/0071R: CAGACATCTTAGCAAACTCTGG, 0072F: CCAGATAGAGCTCCAGATGC/0072R: CGATCTACTTAATCTTACCAGTG; *dhs-28*: 0073F: CATGAGAAGACCACAGCGTAGTC/0067R2: GTACTTTGCACCTTCTTGGGCGAG, 0068F: CAACTACGCTGCTGCCAAAAG/0073R: GTTGGGTCAGCCTTCACACC, 0074F: CAGAGTGCTAGACTTGTCTCAATC/0069R: TGAACGCTTCTG TCTGTTTAC; *daf-22*: 0075F: GGTCTATGTACCTTCAAGTACCG/0075R: CGACGAAATTAAATTAGAGATGG, 0076F2: GCTCTTTCTGTAGACAAACAC/0076R2: CCACTTTGAGAGCGTTTCAG; *prx-10*: 0047F2: CCCTTTAGATAGACTTTTGCTG/0047R: CCTGTTGTGTAATTGGATTATTCTC, 0048F: GAGAATAATCCAATTACACAACAGG/0048R: CCAAAATGAGACTATCCACTTAACG, 0049F: ACCCAGACGCGAATTATTACAGTAG/0049R: CCAGTGAAATATATTGTTGGATGAG

### Genetic mapping

Genetic mapping was conducted essentially in the same way as that described in Davis *et al.* (2005). Briefly, *drop* mutants were crossed with the Hawaiian strain CB4856. F2 progeny with supersized LDs were picked out and tested for homologous recombination events that brought Hawaiian single nucleotide polymorphisms (SNP) into N2 Bristol background.

### Construction of double mutants of drop with acs-22 or dgat-2

We constructed the following double mutants: *drop-1(ssd9)*; *acs-22(tm3236)*, *drop-2(ssd14)*; *acs-22(tm3236)*, *drop-3(ssd36)*; *acs-22(tm3236)*, *acs-22(tm3236) drop-4(ssd206)*, *acs-22(tm3236) drop-5(ssd72)*, *dgat-2(ssd90)*; *drop-6(ssd73)*, *drop-7(ssd75)*; *acs-22(tm3236)*, *drop-8(ssd89)*; *acs-22(tm3236)*, and *drop-9(ssd213)*; *acs-22(tm3236)*. For constructing double mutants, *drop* mutants were mated with *acs-22(tm3236)* or *dgat-2(ssd90)*; at least eight F2s with supersized LDs were picked out and singled to produce F3s. F2s were subjected to *acs-22/dgat-2* genotyping. If F2s were *acs-22* homozygous, *i.e*., *drop/drop*; *acs-22/acs-22*, F3s derived from the F2 were kept as double mutants. The *drop* mutant was inferred as ACS-22/DGAT-2-independent. If none of the F2s was *acs-22/dgat-2* homozygous but heterozygous, F3s derived from F2 heterozygotes were counted to calculate the ratio of supersized LD animals. If the ratio was three quarters, the *drop* mutant was inferred as ACS-22/DGAT-2-dependent. F3s without supersized LDs were singled to produce F4s, and were then subjected to *acs-22/dgat-2* genotyping. F4s of *acs-22/dgat-2* homozygous F3s were kept as double mutants.

### Brood size test

Five stage L4 animals (P0s) of each *drop* reference allele were singled to lay eggs. Each P0 was transferred onto a new plate every 24 hr to lay eggs. Each P0 was allowed to lay eggs for a total of 144 hr when no more eggs were produced. The number of F1 progeny that eventually developed to L4 stage was counted.

### Fasting

Fasting was conducted essentially as in Zhang *et al.* (2010a).

### LD purification and western blot

Purification of LDs was essentially as in Zhang *et al.* (2010b) and Zhang *et al.* (2012) with some modifications. Briefly, 2 × 10^5^ freshly hatched wild-type or *hjSi56* animals were grown on 20 15 cm-NGM/OP50 plates. Animals were allowed to grow to the 1-d adult stage, then harvested and washed three times with 1X PBS/0.001% Triton X-100 (PBST). Animals were then washed with 5 ml buffer A [25 mM Tricine, pH 7.6, 250 mM sucrose, one protease inhibitor cOmplete Tablet (#04693132001, Roche) per 50 ml volume]. Animals were then resuspended in 3 ml buffer A and homogenized by 75 strokes using a dounce homogenizer. Each homogenate was transferred into a 15 ml centrifuge tube. Buffer A was added to the tube to bring the total volume to 10 ml. Homogenate was centrifuged at 1000 *g* for 10 min at 4°. The supernatant was postnuclear supernatant (PNS), from which 9 ml was loaded into a 13 ml centrifuge tube. A total of 3 ml buffer B (20 mM HEPES, pH 7.4, 100 mM KCl, and 2 mM MgCl_2_) was gently added on top of the 9 ml sample. The sample was then centrifuged in a Beckman SW41 Ti rotor at 12,348 *g* for 1 hr at 4°. The LD fraction (the top layer) was carefully collected and washed with 200 µl buffer B and centrifuged at 15,000 *g* for 3 min at 4°, three times in total. A 2 ml fraction in the lower phase of the tube was collected, transferred into a new tube, and centrifuged in a TLS-55 rotor at 100,000 *g* for 1 hr at 4°. The pellet at the bottom of the tube was the total membrane (TM) fraction and was washed three times with buffer B. For western blot, LD protein and TM protein were extracted. Briefly, 1 ml acetone was added to the LD or TM sample. Samples were vortexed rigorously and then chilled at −20° for 1 hr. Samples were centrifuged at 16,000 *g* for 10 min. LD protein and TM protein were spun down as pellets. Pellets were air dried and were dissolved in 30 µl 2X SDS protein sample buffer. Protein concentrations were measured using a BCA kit (#23227, Thermo Scientific). A total of 10 µg of each protein sample was loaded and separated on 8% SDS-PAGE according to standard methods. Proteins separated were subjected to western blot using a mouse anti-FLAG antibody (#F3165-1MG, Sigma-Aldrich) at 1:500 dilution.

### Data availability

Strains and data are available upon request. The authors state that all data necessary for confirming the conclusions presented in the article are represented fully within the article.

## Results

### Design of the screen

**Figure 1:**
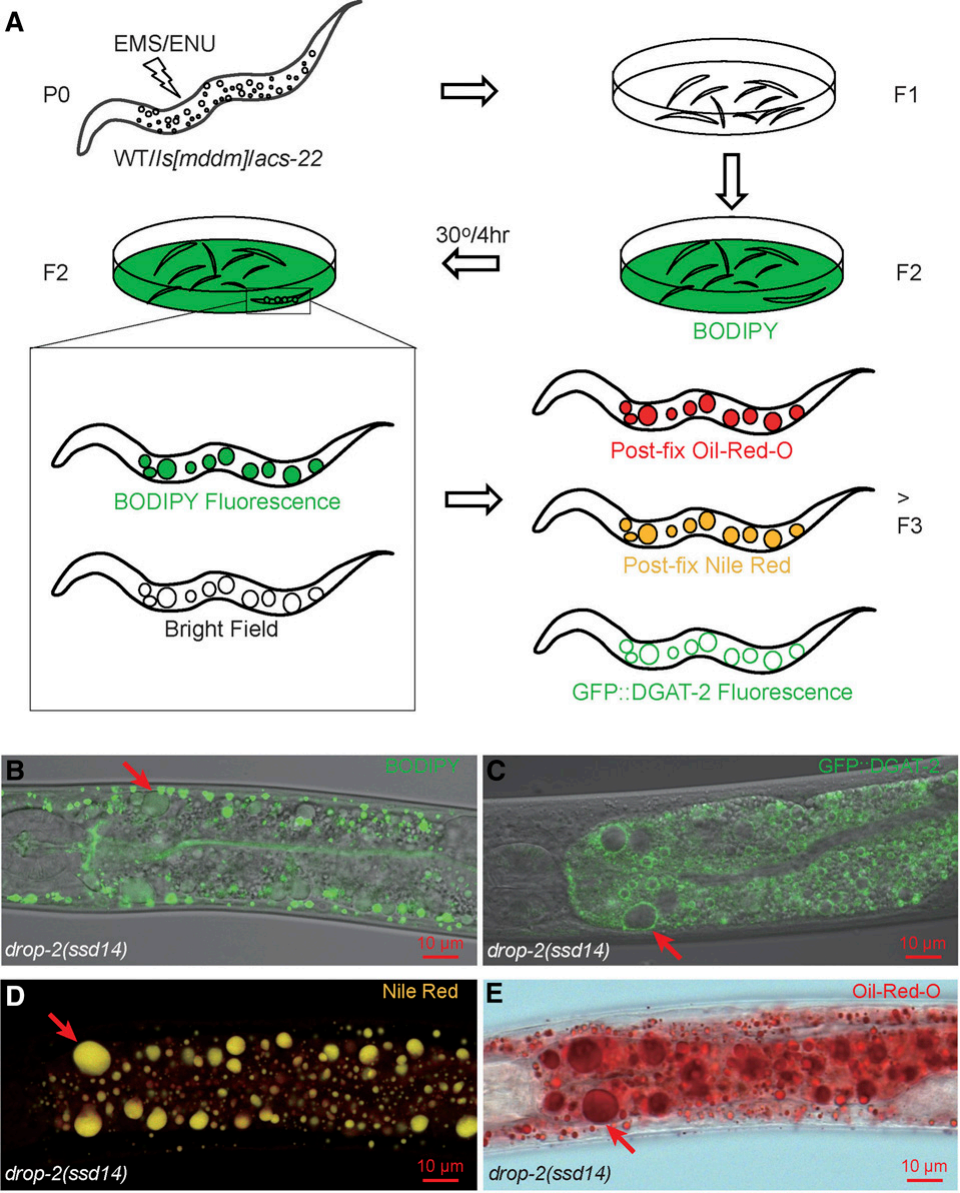
The design of mutagenesis and LD labeling. (A) Both EMS and ENU were used to mutagenize wild type (WT), transgenic lines *ssdIs1* and *ssdIs5[maoc-1*; *dhs-28*; *daf-22*; *mrfp*::*pts1]* (*Is[mddm]*), and *acs-22(tm3236)* P0 animals. F2 progeny were synchronized and transferred onto NGM/OP50 plates layered with BODIPY and were allowed to grow to adulthood at 15° or 20° before being shifted to 30° for 4 hr. Candidate mutants were isolated by virtue of BODIPY-positive and light-refracting globular structures visible under a fluorescence stereoscope at 180× magnification. F3s and further generations of F2 isolates were then tested by postfix Oil-Red-O staining, postfix Nile Red staining, and were crossed into *hjSi56[gfp*::*dgat-2]* for testing GFP::DGAT-2 marking. Supersized LDs (arrows) in *drop-2(ssd14)*, one of the 118 isolates, were readily visible in bright field and were labeled by vital BODIPY (B), by GFP::DGAT-2 (C), by postfix Nile Red with a true color of gold (D), and by postfix Oil-Red-O (E). (B) and (C) are channel mode confocal microscopy images; (D) lambda mode confocal microscopy, also note the diffuse red fluorescence of membrane structures labeled by postfix Nile Red; (E) wide field microscopy.

The main aim of this study was to conduct a large-scale screen for recessive loss-of-function mutants with supersized LDs in *C. elegans*. We extended a previous screen by exploiting several innovations. First, in addition to using the wild-type N2 as a mutagenesis background, we used an integrated stable transgenic line (termed *Is[mddm]*) bearing multiple copies of wild-type *maoc-1*, *dhs-28*, *daf-22*, and a peroxisome import marker *mrfp*::*pts1* cDNAs driven by an intestine-specific *vha-6* promoter. This line was used to bypass the reisolation of *maoc-1*/*dhs-28*/*daf-22*, to immediately identify *prx-10* based on the mislocalization of peroxisome marker protein mRFP::PTS1 (Zhang *et al.* 2010a), and to enrich new genes. Second, since the supersized LD phenotype of *maoc-1*/*dhs-28*/*daf-22*/*prx-10* is a result of enhanced LD growth mediated by the ACS-22-DGAT-2 enzyme complex (Xu *et al.* 2012), we used a deletion mutant *acs-22(tm3236)* as a mutagenesis background in hope of enriching new genes representing novel models of supersized LD formation. Third, before phenotype screening we shifted the F2 progeny from a low cultivation temperature of 15° or 20° to 30° for the brief time of 4 hr, in hope of finding temperature-sensitive (ts) mutants with a fast response to temperature shift. We failed to find any ts mutants with a shift to 25° so we switched to 30°. Fourth, we used ENU as a mutagen in addition to EMS. ENU has a broader mutagenic spectrum of DNA base substitution and fragment deletion (Anderson 1995; De Stasio *et al.* 1997; De Stasio and Dorman 2001). ENU may increase the chance of isolating genes with very low allele frequency, especially those rare ts alleles. In total we conducted six series of screens: EMS/WT, ENU/WT, EMS/*Is[mddm]*, ENU/*Is[mddm]*, EMS/*acs-22*, and ENU/*acs-22*. Each series allowed both non-ts and ts mutant isolation (Figure 1A). Fifth, in all series except one, we increased the haploid genome number to ∼1.0 × 10^5^. The total number of haploid genomes screened was more than half a million (Table 1).

**Table 1:** Isolation origins of the 13 complementation groups and 118 alleles

Group	ts	Alleles	Mutagen/Background (Haploid Genomes)					
			EMS/WT	ENU/WT	EMS/*Is[mddm]*	ENU/*Is[mddm]*	EMS/*acs-22*	ENU/*acs-22*
			(1.18 × 10^5^)	(1.27 × 10^5^)	(0.95 × 10^5^)	(0.32 × 10^5^)	(1.49 × 10^5^)	(1.49 × 10^5^)
*maoc-1 II*		**5**	3	2	x	x	x	x
*dhs-28 X*		**15**	9	6	x	x	x	x
*daf-22 II*		**23**	18	5	x	x	x	x
*prx-10 III*		**2**			2		x	x
*drop-1 II*	ts (38/38)	**38**	5	4	2	2	16	9
*drop-2 IV*		**22**	5	5	12		x	x
*drop-3 IV*	ts (5/5)	**5**	3			2	x	x
*drop-4 X*		**1**						1
*drop-5 X*	ts (2/2)	**2**	1		1		x	x
*drop-6 X*	ts (2/2)	**2**		2			x	x
*drop-7*		**1**		1			x	x
*drop-8 III*	ts (1/1)	**1**						1
*drop-9 II*		**1**	1				x	x
Total		**118**	**45**	**25**	**17**	**4**	**16**	**11**

The 13 complementation groups consist of four known groups (*maoc-1*, *dhs-28*, *daf-22*, and *prx-10*), which are separated from the nine new *drop* groups. Six screen series are defined according to the mutagen and background used. WT, N2 Bristol. *Is[mddm]*, *ssdIs1*, and *ssdIs5[maoc-1*; *dhs-28*; *daf-22*; *mrfp*::*pts1]*. *acs-22*, a deletion allele *acs-22(tm3236)*. Numbers of alleles are listed. ts, temperature-sensitive alleles; x, no mutant of the group expected due to *Is[mddm]* bypassing or *acs-22* suppression; empty cell, no allele isolated.

We used five labeling and visualization criteria to identify supersized LDs: vital BODIPY staining, bright field visualization, postfix Oil-Red-O staining, postfix Nile Red staining (Zhang *et al.* 2010a,b), and GFP::DGAT-2 LD marker labeling (Xu *et al.* 2012). To test whether GFP::DGAT-2 specifically labels LDs, we purified LDs and TM from *hjSi56[vha-6p*::*3xflag*::*gfp*::*dgat-2]* transgenic animals, and found that DGAT-2 localizes to LD but not to TM (Supplemental Material, Figure S1A). Under lambda mode confocal microscopy, LRO’s autofluorescence appears as a filled globe, while LD’s GFP::DGAT-2 fluorescence appears as an encircled globe. The two kinds of fluorescence display distinct emission spectra and the globular structures labeled by each completely exclude the other (Figure S1, B–E). *glo-4* mutation eliminates LROs, leaving fluorescent structures 100% GFP::DGAT-2 positive (Figure S1, F and G). These data corroborated the specificity of GFP::DGAT-2 as an LD marker. In wild type, 78.9% of vital BODIPY-labeled structures are encircled by GFP::DGAT-2, while 21.1% are not and should be LROs since *glo-4* mutation leaves BODIPY structures 99.9% encircled by GFP::DGAT-2 (Figure S2, A–C).

To screen for recessive mutants, F2 progeny of mutagenized P0s were grown on BODIPY-layered NGM/OP50 media. F2s with supersized LDs were picked out by virtue of BODIPY fluorescence and bright light-refraction under a stereoscope at 180× magnification. Among the isolates picked out from the six screening series, only 118 (about a third of total) survived and proliferated. F3 and further generations of these 118 isolates were subjected to postfix Oil-Red-O staining, postfix Nile Red staining, and GFP::DGAT-2 labeling (Figure 1A) which unsurprisingly confirmed all 118 as *bona fide* supersized LD mutants (*e.g.*, Figure 1, B–E), a further validation of each LD labeling and visualization method.

### Identification of nine novel complementation groups of supersized LD mutants

The 118 isolates were backcrossed four times, segregated from the original genetic background when necessary, and examined for temperature sensitivity. During crossing, we found that all mutants are recessive loss-of-function, completely penetrant, and with no detectable maternal effect. These isolates were tested for complementation first against known *maoc-1*, *dhs-28*, *daf-22*, and *prx-10* alleles. We found five *maoc-1* alleles, 15 *dhs-28*, 23 *daf-22*, and two *prx-10*. The other 73 were tested against each other and sorted into nine novel complementation groups named *drop* (lipid droplet abnormal) (Table 1). The isolation origins of these 13 complementation groups display expected patterns. *maoc-1*, *dhs-28*, and *daf-22* were bypassed in the EMS/ENU/*Is[mddm]* and EMS/ENU/*acs-22* series; *prx-10* was bypassed in the EMS/ENU/*acs-22* series. Interestingly, six of the nine *drop* groups, *drop-2*, *3*, *5*, *6*, *7*, and *9*, were also bypassed in the EMS/ENU/*acs-22* series. The temperature regimen proved a success: five of the nine groups, *drop-1*, *3*, *5*, *6*, and *8*, are ts. Curiously, the temperature sensitivity is either all or none: all alleles of *drop-1*, *3*, *5*, *6* and *8* are ts; all alleles of *drop-2*, *4*, *7*, *9*, *maoc-1*, *dhs-28*, *daf-22*, and *prx-10*, are non-ts. The restrictive temperature of *drop-1*, *3*, *6*, and *8* is 30° as was screened for, while that of *drop-5* (20°) occurred fortuitously.

**Figure 2:**
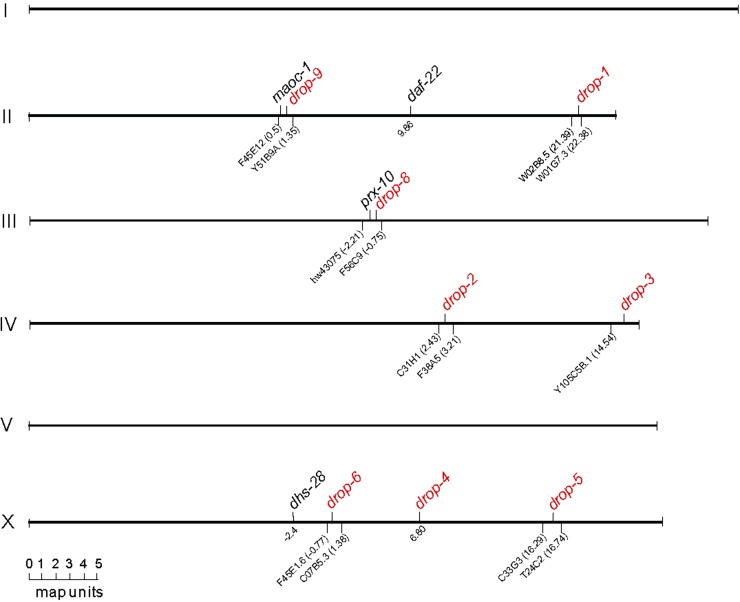
Positions of the eight *drop* loci. The eight loci (red) except *drop-4* were mapped by SNP-based chromosome mapping and interval mapping. *drop-4* was cloned as *sams-1* and its genetic position is at X 6.80. Each of the other seven *drop* loci was positioned by the two closest encompassing SNPs underpinning. Genetic positions of these SNPs were extrapolated from wormbase.org and were located onto each linkage group/chromosome to scale. The four known genes, *maoc-1 II*, *dhs-28 X*, *daf-22 II*, and *prx-10 III*, were positioned accordingly for reference.

Heterozygotes of the only allele of *drop-7* segregated 1/16 supersized LD progeny (see later), indicating mutations in two unlinked genetic loci. Heterozygotes of the other eight *drop* groups segregated 1/4 supersized LD progeny, indicating mutation in a single locus. Seven of the eight single *drop* loci were positioned by SNP-based chromosome mapping and three-point interval mapping (Davis *et al.* 2005) (Figure S3); the other locus *drop-4* was cloned by direct sequencing. The eight single loci reside in discrete short physical regions of Chr II, III, IV, and X (Figure 2).

### Class I (drop-5 and drop-9) is up-regulated in LD growth and defective in peroxisome import

**Figure 3:**
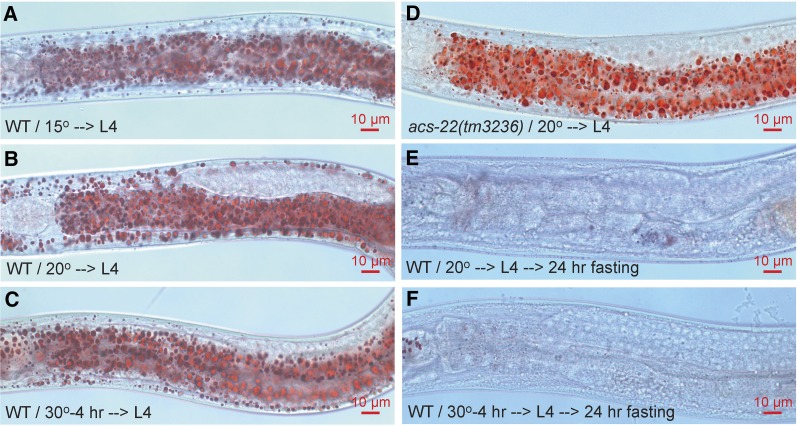
Wild-type LDs are small in size and liable to fasting-induced hydrolysis. (A) and (B) WT animals were grown continuously at 15° or 20° to late L4 stage. Animals were fixed, stained with Oil-Red-O, and imaged on a compound microscope. LDs are usually smaller than 3 μm in diameter at stage L4. (C) WT animals grown continuously at 20° to early/mid L4 stage were then shifted to 30° for 4 hr to reach late L4. LD size with this temperature shift is not obviously different to that with continuous growth at 15° or 20°. (D) LD size in *acs-22(tm3236)* mutant is indifferent to that in WT. (E) and (F) WT animals were grown continuously at 20° or shifted to 30° for 4 hr to reach late L4 stage. These animals were then fasted in M9 buffer at 20° for 24 hr. LDs in these animals are almost completely hydrolyzed. In each Oil-Red-O experiment, > 50 stained animals were examined under a stereoscope, and at least 20 animals were imaged on a compound microscope. In other figures, if not otherwise indicated, all staining was conducted on late stage L4 animals, fasting was at 20°, the same numbers of Oil-Red-O-stained animals were examined and imaged, and images are anterior half of the animal with head to the left.

**Figure 4:**
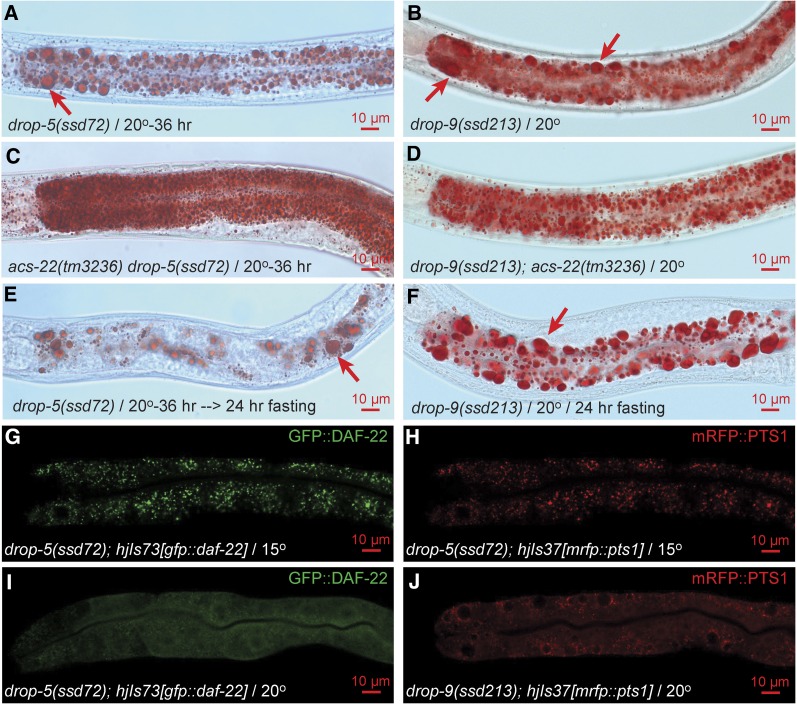
Supersized LD phenotype and peroxisome import defect of class I mutants *drop-5* and *drop-9*. *drop-5(ssd72)^ts^* animals grew supersized LDs (arrow) when shifted from the permissive temperature of 15° to the restrictive temperature of 20° for 36 hr (A). Supersized LDs were abolished by *acs-22* null mutation (C). Supersized LDs were partially resistant to fasting-induced hydrolysis (E). The non-ts mutant *drop-9(ssd213)* is similar to *drop-5(ssd72)^ts^* in supersized LD phenotype, dependence on ACS-22, and resistance to hydrolysis (B, D, and F). At the permissive temperature of 15°, GFP::DAF-22 and mRFP::PTS1 proteins in *drop-5(ssd72)^ts^* are imported into peroxisomes, displaying a granular pattern (G and H); at 20°, GFP::DAF-22 is not imported, displaying a diffuse pattern (I). mRFP::PTS1 is not imported in *drop-9(ssd213)* (J). For (G–J), > 50 animals were examined under fluorescence stereoscope, and at least five typical animals were imaged on confocal microscope.

As indicated by isolation origins, the *drop* mutants may be classified according to whether they are dependent on ACS-22 for supersized LD phenotype and whether they affect peroxisome marker mRFP::PTS1 import. Consistent with previous reports (Zhang *et al.* 2010a,b), wild-type LDs are usually below 3 μm in diameter at stage L4 and display no obvious size difference when grown continuously at 15°, 20°, or shifted to 30° for a brief period of 4 hr (Figure 3, A–C). At a restrictive temperature of 20°, the reference allele *ssd72^ts^* of *drop-5* accumulates supersized LDs > 3 μm in and only in the intestinal cells throughout the anterior–posterior axis (Figure 4A). The only allele of *drop-9*, *ssd213*, is a non-ts mutant with intestinal supersized LDs formed at all three temperatures (Figure 4B; unpublished results). The *acs-22(tm3236)* null mutation has no effect on wild-type LDs (Xu *et al.* 2012) but completely prevents supersized LD formation in *drop-5(ssd72)^ts^* and *drop-9(ssd213)* (Figure 3D and Figure 4, C and D). Supersized LDs that have already formed in *drop-5(ssd72)^ts^* and *drop-9(ssd213)* are mostly resistant to hydrolysis induced by a 24 hr-fasting post L4 while LDs in wild type are not (Figure 3, E and F and Figure 4, E and F). *drop-5(ssd72)^ts^* was isolated in the EMS/*Is[mddm]* series and was noted for its ts peroxisome import defect: peroxisome matrix protein GFP::DAF-22 and mRFP::PTS1 are correctly imported at 15° but not at 20° (Figure 4, G–I). Similarly, mRFP::PTS1 is not imported in *drop-9(ssd213)* (Figure 4J). Thus, *drop-5* and *drop-9* are similar to *maoc-1/dhs-28/daf-22/prx-10* in supersized LD phenotype, ACS-22-dependence, and resistance to fasting-induced hydrolysis, and similar to *prx-10* in peroxisome import defect (Zhang *et al.* 2010a; Xu *et al.* 2012). It strongly suggests that *drop-5* and *drop-9* supersized LDs form by an enhancement of LD growth and an inhibition of LD hydrolysis. *drop-5* and *drop-9* may encode peroxins and are categorized as class I.

### Class II (drop-2, drop-3, drop-6 and drop-7) is up-regulated in LD growth but not defective in peroxisome import

**Figure 5:**
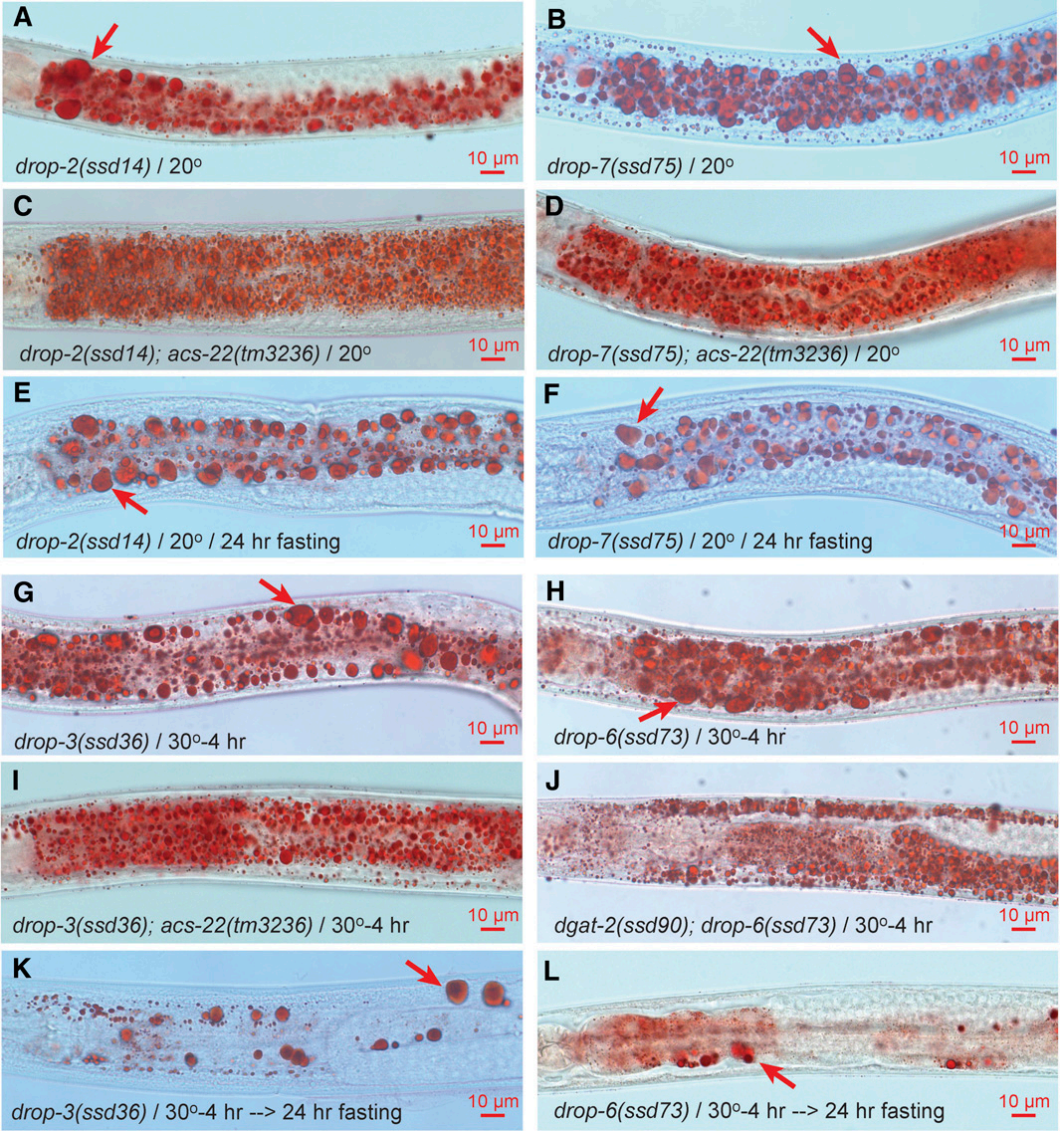
Supersized LD phenotype of class II mutants *drop-2*, *drop-3*, *drop-6*, and *drop-7*. Non-ts mutants *drop-2(ssd14)* and *drop-7(ssd75)* grow supersized LDs (A and B). The supersized LDs are dependent on ACS-22 (C and D) and are resistant to hydrolysis (E and F). ts mutants *drop-3(ssd36)^ts^* and *drop-6(ssd73)^ts^* grow supersized LDs after being shifted to the restrictive temperature of 30° for 4 hr (G and H). The supersized LDs are dependent on ACS-22/DGAT-2 (I and J) and are partially resistant to hydrolysis (K and L).

Class II mutants *drop-2*, *drop-3*, *drop-6*, and *drop-7* are similar to class I in supersized LD phenotype, ACS-22/DGAT-2-dependence, and resistance to fasting-induced hydrolysis (Figure 5, A–L). But these four groups are not peroxisome import defective. Interesting differences exist between the four groups. All 22 alleles of *drop-2* are non-ts and exhibit similarly supersized LDs. It is unlikely that these 22 alleles are a series of variable partial loss-of-function mutations since *drop-2(ssd29)* is an amber mutant suppressible by *sup-7(st5)*. All five alleles of *drop-3* and both alleles of *drop-6* are ts with a restrictive temperature of 30°. Furthermore, the supersized LDs formed at 30° for 4 hr are only partially resistant to fasting-induced hydrolysis (Figure 5, K and L). The unusually high restrictive temperature and the partial resistance to fasting-induced hydrolysis may be due to the existence of *drop-3* and *drop-6* homologs. Consistent with this notion is that *ssd75*, the only *drop-7* allele, is non-ts and is fully resistant to fasting-induced hydrolysis (Figure 5, B and F). *drop-7(ssd75)* appears mutated in two unlinked genes since its heterozygote segregates ∼1/16 (9/137) supersized LD progeny. However, *drop-3* and *drop-6* are unlikely to be homologous to each other since the double mutant *drop-3(ssd36)^ts^*; *drop-6(ssd73)^ts^* is not stronger than each single mutant in LD phenotype. With a similarity to *maoc-1/dhs-28/daf-22* in supersized LD phenotype and a lack of peroxisome import defect, *drop-2*, *drop-3*, *drop-6*, and *drop-7* may encode additional enzymes of peroxisomal fatty acid β-oxidation and are thus categorized as class II.

### Class III (drop-1 and drop-8) forms supersized LDs by fusion

**Figure 6:**
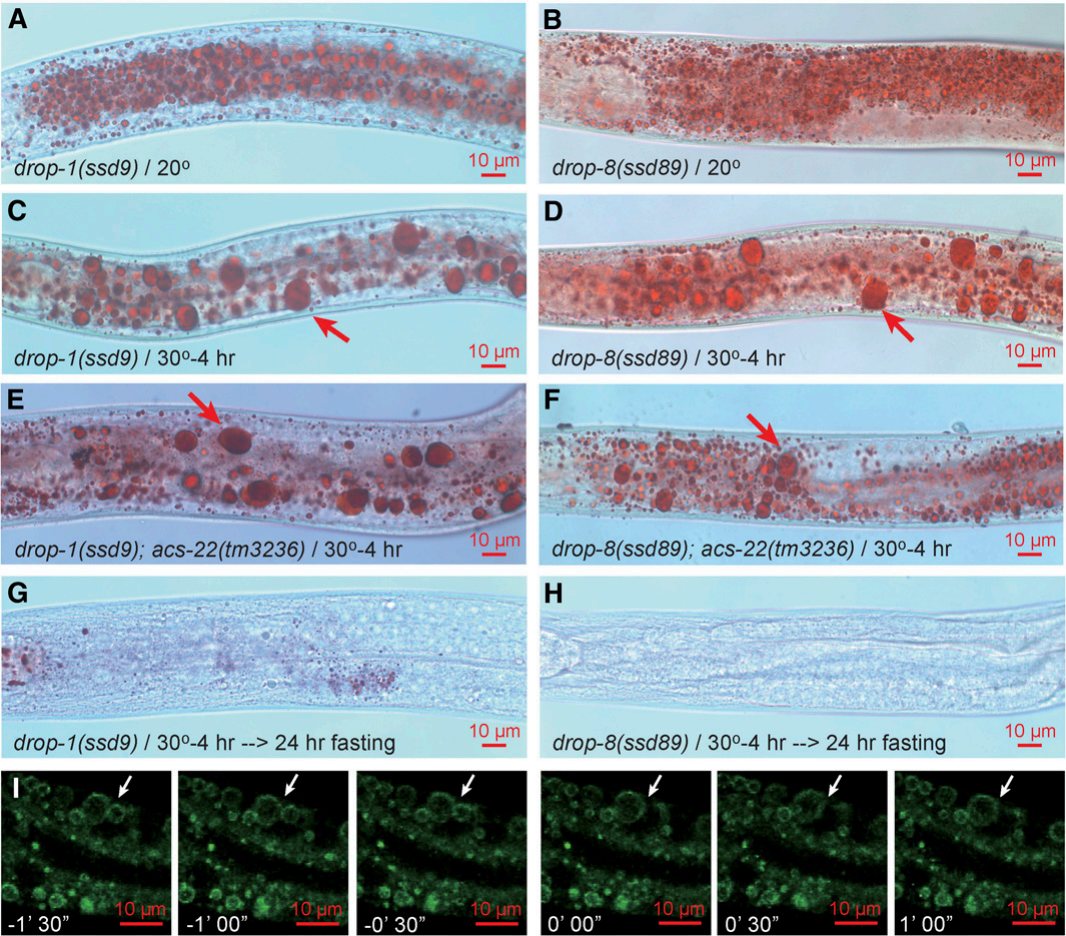
Supersized LD phenotype of class III mutants *drop-1* and *drop-8*. *drop-1(ssd9)^ts^* and *drop-8(ssd89)^ts^* animals form supersized LDs after being shifted from the permissive temperature of 20° to the restrictive temperature of 30° for 4 hr (A–D). The formation of supersized LDs is accompanied by an obvious disappearance of small LDs (note the difference between A and C, B and D). Supersized LDs are not dependent on ACS-22 (E and F) and are almost completely hydrolyzed by fasting (G and H). To visualize LD fusion, *hjSi56[gfp*::*dgat-2]* was crossed into *drop-1(ssd2)^ts^*. L4 animals were mounted on a confocal microscope and subjected to 30° heat treatment. Time-lapse 3-D confocal fluorescence imaging was conducted. As shown in one example, two LDs (white arrow) fused in a short time window of < 30 sec to form a supersized LD > 5 μm in diameter (I). Each image is an extended focus view of a 9 μm Z-stack. *n* > 20.

All alleles of class III mutants *drop-1* and *drop-8* are ts with a restrictive temperature of 30° (Figure 6, A–D). This class differs drastically from class I and II mutants in three aspects. First, the supersized LD phenotype is not abolished by *acs-22* mutation (Figure 6, E and F). Second, supersized LDs are completely exhausted by fasting-induced hydrolysis (Figure 6, G and H). Third, the formation of supersized LDs upon a shift to 30° for 4 hr is accompanied by an obvious decrease of small LDs, indicating a process of enhanced LD fusion (Figure 6, A–D). To directly test for LD fusion, we time-lapse imaged GFP::DGAT-2-labeled LDs in *drop-1* upon an up-shift to 30°. More than 20 fusion events were recorded. As shown in one example, two LDs directly fuse during a short time window of 30 sec to form a supersized LD with a diameter > 5 μm (Figure 6I).

### Class IV (drop-4) forms supersized LDs independent of ACS-22 and is hydrolysis-resistant

**Figure 7:**
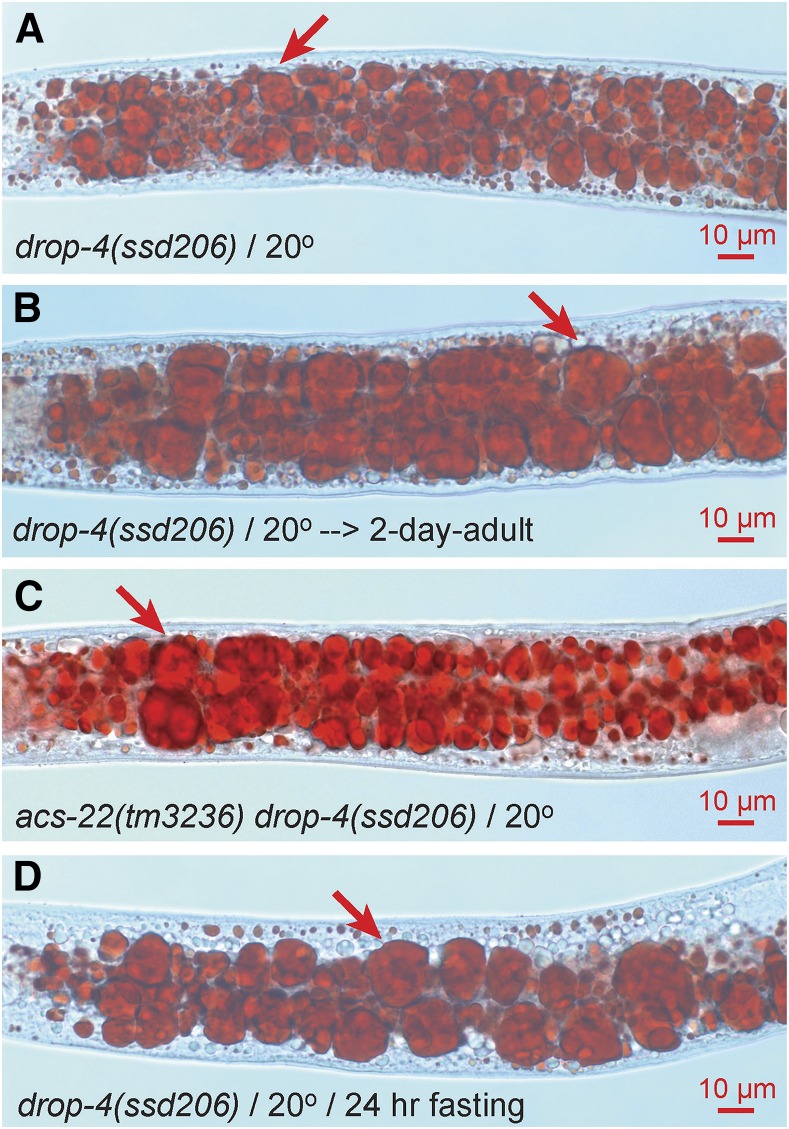
Supersized LD phenotype of class IV mutant *drop-4(ssd206)*. The non-ts mutant *drop-4(ssd206)* forms supersized LDs (A). Supersized LDs can reach up to 20 μm in diameter at 2-d adult stage (B). Supersized LDs are not dependent on ACS-22 and are resistant to hydrolysis (C and D).

Class IV is defined by a single group with a single non-ts allele *drop-4(ssd206)*. The supersized LD phenotype of *drop-4(ssd206)* is already pronounced at L4 stage and increases to a phenomenal level at 2-d adult stage (Figure 7, A and B). The extremely large LDs resemble those in a previously reported mutant *sams-1* (Li *et al.* 2011). *drop-4(ssd206)* does not complement a deletion mutant *sams-1(ok2946)* and bears a missense mutation Ala42Val at the SAMS-1 protein level. So, *drop-4* is *sams-1*. Unlike in *drop-1* and *drop-8*, the formation of supersized LDs in *drop-4*/*sams-1* is unaccompanied by a detectable clearance of small LDs (Figure 7A), independent of ACS-22, and resistant to fasting-induced hydrolysis (Figure 7, C and D). These features make *drop-4*/*sams-1* distinct from class I, II, and III.

### Allele frequency, enrichment of novel genes, and a test of saturation

In the six series of genetic screens we isolated 13 complementation groups. Four are the same peroxisome mutants as previously found. Nine are novel. This collection of mutants showed a drastic difference in allele numbers. Four genes (*dhs-28*, *daf-22*, *drop-1* and *drop-2*) are defined by > 15 alleles; the other four (*drop-4*, *drop-7*, *drop-8*, and *drop-9*), by just one (Table 1). To estimate the screening efficiency, we calculated allele frequencies of these genes according to their isolation origins (Table 2).

**Table 2:** Allele frequencies with two different mutagens and three backgrounds

	Mutagen		Background		
	EMS	ENU	WT	*Is[mddm]*	*acs-22*
*maoc-1 II*	2.5 × 10^−5^*^a^*	1.6 × 10^−5^*^a^*	2.0 × 10^−5^		
*dhs-28 X*	7.6 × 10^−5^*^a^*	4.7 × 10^−5^*^a^*	6.1 × 10^−5^		
*daf-22 II*	15.3 × 10^−5^*^a^*	3.9 × 10^−5^*^a^*	9.4 × 10^−5^		
*prx-10 III*	0.9 × 10^−5^*^b^*			1.6 × 10^−5^	
*drop-1 II*	6.4 × 10^−5^*^c^*	4.9 × 10^−5^*^c^*	3.7 × 10^−5^	3.1 × 10^−5^	8.4 × 10^−5^
*drop-2 IV*	8.0 × 10^−5^*^b^*	3.1 × 10^−5^*^b^*	4.1 × 10^−5^	9.4 × 10^−5^	
*drop-3 IV*	1.4 × 10^−5^*^b^*	1.3 × 10^−5^*^b^*	1.2 × 10^−5^	1.6 × 10^−5^	
*drop-4 X*		0.3 × 10^−5^*^c^*			0.3 × 10^−5^
*drop-5 X*	0.9 × 10^−5^*^b^*		0.4 × 10^−5^	0.8 × 10^−5^	
*drop-6 X*		1.3 × 10^−5^*^b^*	0.8 × 10^−5^		
*drop-7*		0.6 × 10^−5^*^b^*	0.4 × 10^−5^		
*drop-8 III*		0.8 × 10^−5^*^c^*			0.3 × 10^−5^
*drop-9 II*	0.5 × 10^−5^*^b^*		0.4 × 10^−5^		
Total	**43.5 × 10^−5^**	**22.5 × 10^−5^**	**28.5 × 10^−5^**	**16.5 × 10^−5^**	**9.0 × 10^−5^**

a The number of alleles divided by the number of haploid genomes of the WT screen since *Is[mddm]* transgene bypasses the selection of and *acs-22* suppresses these mutants. In a similar EMS/WT screen in Zhang *et al.* (2010) and Butcher *et al.* (2009), allele frequency of *maoc-1* is 5.6 × 10^−5^; *dhs-28*, 11.1 × 10^−5^; *daf-22*, 11.1 × 10^−5^; *prx-10*, 2.7 × 10^−5^.

b The number of alleles divided by the sum of haploid genomes of the WT and *Is[mddm]* screens since *acs-22* suppresses these mutants.

c The number of alleles divided by the sum of haploid genomes of the WT, *Is[mddm]*, and *acs-22* screens since *acs-22* does not suppress these mutants.

We first compared the efficiency of the two mutagens. In the EMS/WT screen, allele frequencies of three known genes are comparable to those reported before: 2.5 × 10^−5^ *vs.* 5.6 × 10^−5^ of *maoc-1*, 7.6 × 10^−5^ *vs.* 11.1 × 10^−5^ of *dhs-28*, and 15.3 × 10^−5^ *vs.* 11.1 × 10^−5^ of *daf-22* (Butcher *et al.* 2009; Zhang *et al.* 2010a). ENU mutagenesis did not result in a higher allele frequency for these three genes. The cumulative allele frequency of all 13 genes with ENU is 22.5 × 10^−5^, lower than 43.5 × 10^−5^ with EMS. For every one of the nine genes (*maoc-1*, *dhs-28*, *daf-22*, *prx-10*, *drop-1*, *drop-2*, *drop-3*, *drop-5*, and *drop-9*), the frequency with ENU is consistently lower than that with EMS and this difference is significant (*P* = 0.05, paired two-sample *t*-test). However, *drop-4*, *drop-6*, *drop-7*, and *drop-8* with only one or two rare alleles were isolated only from ENU mutagenesis (Table 2), suggesting that, although less efficient than EMS, ENU may be suitable for isolating genes with rare alleles due to its ability to target a broader spectrum of base pairs (Anderson 1995; De Stasio *et al.* 1997). Consistent with this speculation is that for the 45 sequenced mutation sites of *maoc-1*, *dhs-28*, *daf-22*, and *prx-10*, mutations by EMS are 93.7% GC→AT transition, 3.1% GC→TA transversion, 3.1% AT→CG transversion, while mutations by ENU are 61.5% GC→AT transition, 7.7% GC→TA transversion, 7.7% AT→GC transition, 15.4% AT→TA transversion, and 7.7% insertion-deletion (Indel) (Table S1).

We next assessed the efficiency of using *Is[mddm]* and *acs-22* as mutagenesis backgrounds in enriching new genes or enriching new alleles. Indeed, the *Is[mddm]* and *acs-22* series realized the goal of not only bypassing known genes but also enriching new genes or new alleles. For example, the *Is[mddm]* series completely bypass *maoc-1*/*dhs-28*/*daf-22* and isolate *prx-10*/*drop-2*/*drop-3*/*drop-5* at moderately higher allele frequencies than the WT series. According to our phenotype classification criteria, *maoc-1*/*dhs-28*/*daf-22/prx-10* and *drop-2*/*drop-3*/*drop-5* belong to the LD growth enhancement model common to class I and class II. It suggests that the bypassing strategies may be particularly useful to enrich new genes of the same phenotype/function class or a new gene class. In agreement with this deduction, class III mutant *drop-1* is not enriched in the nonclass-bypassing *Is[mddm]* series but is enriched in the class I and II-bypassing *acs-22* series with an allele frequency 2.27-fold of that in the WT series. Class III mutant *drop-8* and class IV *drop-4*/*sams-1* are enriched in the *acs-22* series but unfound in the WT or *Is[mddm]* series (Table 2).

If the mutation probabilities of all 13 genes obey Poisson distribution, the number of unidentified genes may be calculated with the Poisson equation. We calculated the number of unidentified genes in the WT series since it permits the isolation of all four classes. In EMS/WT, ENU/WT, and the combined WT series, no gene was unidentified. In the combined WT/*Is[mddm]*/*acs-22* series, no gene was unidentified (Table 3). We interpret these data as meaning that the screening system was saturated but not necessarily that no new genes will be discovered (see *Discussion*).

**Table 3:** The extent of saturation for genes that can be mutated to a fertile supersized LD phenotype

Series	WT	EMS/WT	ENU/WT	WT + *Is[mddm]*+*acs-22*
*maoc-1 II*	5	3	2	5
*dhs-28 X*	15	9	6	15
*daf-22 II*	23	18	5	23
*prx-10 III*				2
*drop-1 II*	9	5	4	38
*drop-2 IV*	10	5	5	22
*drop-3 IV*	3	3		5
*drop-4 X*				1
*drop-5 X*	1	1		2
*drop-6 X*	2		2	2
*drop-7*	1		1	1
*drop-8 III*				1
*drop-9 II*	1	1		1
*λ*	7.0	5.6	3.6	9.0
*P(0)**^a^*	0.0009	0.0036	0.0281	0.0001
*N(0)**^b^*	0.009	0.029	0.202	0.001

Allele numbers are listed for each gene according to isolation origins. *λ*, mean allele number; *P(0)*, probability of unidentified genes; *N(0)*, number of unidentified genes.

a Assume that the allele frequency observes Poisson distribution. The probability of unidentified genes, *i.e.*, the category with 0 allele, follows *P(κ)* = *λ^κ^*·*e^-λ^/κ!*. When *κ* = 0, *P(0)* = *e^-λ^*.

b *N(0)* = *N*·*e^-λ^/(1-e^-λ^)*. *N*, number of identified genes.

## Discussion

The fact that TAG is stored in LDs in *C. elegans* was not established until Zhang and colleagues analyzed wild type, *glo* mutants, and a class of peroxisomal β-oxidation mutants. They found that: (1) *C. elegans* LDs as visualized by transmission electron microscopy are electron translucent structures delimited by a phospholipid monolayer; (2) LDs are not abolished in *glo* mutants and supersized LDs form in peroxisomal β-oxidation mutants; (3) in wild type, *glo* mutants, and peroxisomal β-oxidation mutants, small and supersized LDs can be labeled both by postfix Oil-Red-O and by postfix Nile Red staining; (4) postfix Nile Red labels LDs and emits fluorescence with a true color of gold; (5) vital Nile Red only labels LROs with a red fluorescence in wild type but labels both LROs and LDs in peroxisomal β-oxidation mutants; (6) vital BODIPY labels LDs in addition to LROs with the same emission fluorescence; and (7) vital BODIPY-labeled LDs can be separated from LROs by density centrifugation-based purification or by genetically ablating LROs with *glo-4* mutation (Zhang *et al.* 2010a,b). These findings explained previous intriguing results that postfix Nile Red staining and postfix Oil-Red-O staining serve as proxies for relative TAG level better than vital Nile Red does (Hermann *et al.* 2005; Schroeder *et al.* 2007; Brooks *et al.* 2009; O’Rourke *et al.* 2009), and identified *C. elegans* fat storage organelles as LDs instead of “fat storing LROs” or “vesicles distinct from LROs”. The dual labeling of LDs and LROs by BODIPY was later confirmed and exploited by independent studies (Klapper *et al.* 2011; Xu *et al.* 2012; Ehmke *et al.* 2014). The specific labeling of LDs by postfix Nile Red and Postfix Oil-Red-O was also exploited (Liang *et al.* 2010; Xie and Roy 2012; Vrablik *et al.* 2015), and density purification of LDs has led to the characterization of LD proteomes (Zhang *et al.* 2012; Vrablik *et al.* 2015).

Postfix Nile Red staining and Oil-Red-O staining, however, can only serve as a semiquantitative measurement for TAG levels for the following reasons. First, Nile Red fluorescence and Oil-Red-O staining intensity are difficult to normalize against body size or protein mass, which usually differ between wild type and various mutants. Second, staining intensities and image acquisition vary to some extent between individuals and between staining trials of the same genotype (this study and our unpublished observations) (Brooks *et al.* 2009; O’Rourke *et al.* 2009). Third, postfix Nile Red gives a diffuse background red fluorescence typical of phospholipids in addition to the gold fluorescence of LDs (Figure 1D) (Greenspan *et al.* 1985; Zhang *et al.* 2010b), making indiscriminate quantification of fluorescence intensity error-prone for TAG levels. The application of vital BODIPY fluorescence intensity to TAG quantification suffers similar problems: in addition to labeling intestinal LDs, BODIPY labels intestinal LROs and hypodermal LDs with a much stronger intensity (Zhang *et al.* 2010a,b). Thus, while these staining methods are well suited for qualitative LD labeling, a precise quantification of TAG in *C. elegans* depends on quantitative lipid analytical chemistry with a normalization against total lipid/phospholipid mass or total protein mass (Schroeder *et al.* 2007; Brooks *et al.* 2009; O’Rourke *et al.* 2009; Zhang *et al.* 2010a).

Although qualitatively specific for LDs, postfix Nile Red and Oil-Red-O are too labor-intensive for large-scale genetic screens. Most transgenic LD protein markers such as ATGL-1::GFP, GFP::DGAT-2, DHS-3::GFP, R01B10.6::GFP; PLIN1::GFP, and DHS-4::GFP are functional lipid metabolism proteins whose transgenic expression levels should be kept low so as not to complicate the LD phenotype (Zhang *et al.* 2010a; Xu *et al.* 2012; Zhang *et al.* 2012; Klemm *et al.* 2013; Liu *et al.* 2014; Vrablik *et al.* 2015). Thus, transgenic LD markers necessitate high-power microscopy which is also labor-intensive. Raman scattering imaging has the same practical limitation in addition to the problem of segregating TAG signal from protein and phospholipid signals (Hellerer *et al.* 2007; Wang *et al.* 2014). The reliability and efficacy of labeling methods are key to the applicability of *C. elegans* to large-scale forward genetic screens for LD mutants, and the success of isolating forward genetic mutants with *bona fide* mutant LD phenotypes demonstrates the reliability and efficacy of these methods – to date, apart from a forward screen of an unknown scale based on R01B10.6::GFP which identified a mini-sized LD mutant (Klemm *et al.* 2013), no transgenic marker, postfix staining, or Raman microscopy-based unbiased forward genetic screen has been reported. Small-scale candidate gene and RNAi screens revealed no known or new supersized LD mutants (Liu *et al.* 2014). Interestingly, several mutants with enlarged LDs in oocytes and embryos were identified by DIC microscopy screening of a collection of candidate lethal mutants and animals subjected to RNAi of a small pool of candidate genes. But these mutants did not form supersized LDs at larval or adult stage (Schmokel *et al.* 2016). In contrast, the vital BODIPY-based large-scale forward genetic screen proved successful in isolating supersized LD mutants here and in our previous, smaller scale study (Zhang *et al.* 2010a).

Our study was successful in realizing two aims: to further address *C. elegans* LD labeling methodology and to explore the full potential of *C. elegans* forward genetic screens to establish models of LD size regulation. When incorporated into supersized LDs, BODIPY emits a strong enough fluorescence which can be readily visualized under a stereo fluorescence microscope, allowing us to rapidly screen millions of F2 animals descended from 0.67 million mutagenized haploid genomes. A complication of vital BODIPY is that it labels LROs in addition to LDs. This problem was obviated by introducing the *glo-4* mutation to eliminate LROs (Figure S1 and Figure S2) (Schroeder *et al.* 2007; Zhang *et al.* 2010b). Even in the wild-type background, the presence of LROs did not pose a problem since we looked for a phenotype of size increase and supersized LRO mutants were not found in this study or in a previous direct RNAi screen of LRO phenotype (Soukas *et al.* 2013). As such, our forward genetic screen for supersized BODIPY-positive structures isolated 118 mutants that were all confirmed as supersized LD mutants by postfix Nile Red staining, postfix Oil-Red-O staining, and GFP::DGAT-2 marking (Figure 1). The BODIPY screen allowed the isolation of four mutant complementation groups reported before, and more importantly, nine novel complementation groups representing four models of LD regulation. One group *drop-4* was cloned as *sams-1*, a supersized LD mutant that was originally identified not by direct LD phenotype screening but by candidate gene approaches based on proteomics and gene expression data (Li *et al.* 2011; Walker *et al.* 2011; Ehmke *et al.* 2014). The success of our forward genetic screen justifies the BODIPY approach. We note that the BODIPY approach may not reveal subtle LD phenotypes such as size decrease, small change in number, and change in distribution pattern, for which screening with transgenic markers or postfix staining might also be required. Nevertheless, we suggest that the supersized LD phenotype may serve as a benchmark for testing the reliability and efficacy of any LD labeling and imaging methods in genetic screens.

Have we explored the full potential of the current BODIPY-based forward genetic screens? We think so for the following considerations. The ease with vital BODIPY made it possible to screen a large number of 6.7 × 10^5^ mutagenized haploid genomes. The use of a novel temperature regimen led to the identification of four genes (*drop-1*, *3*, *6*, and *8*) that would otherwise have escaped isolation. The use of a gene-class-bypassing strategy permitted the identification of two additional genes (*drop-4* and *8*). The use of ENU, a mutagen with a broader mutagenic spectrum (Anderson 1995; De Stasio *et al.* 1997), allowed the identification of four genes (*drop-4*, *6*, *7*, and *8*). In total, we identified 118 *bona fide* supersized LD mutants, of which 73 are mutations in nine new genes (Table 1 and Table 2). How frequent is the occurrence of supersized LD mutants? The total allele frequency induced by EMS is 43.5 × 10^−5^, 4.8 × 10^−5^ per gene, 10 times lower than 5 × 10^−4^ of an average gene (Brenner 1974). The average allele frequency of *maoc-1/dhs-28/daf-22* is 6.6 × 10^−5^, comparable to 7.6 × 10^−5^ in a previous report (Zhang *et al.* 2010a). This suggests that the average allele frequency of supersized LD mutants is inherently low. We used the Poisson equation to estimate whether our screen system was saturated, *i.e.*, whether additional genes were unidentified. As shown in Table 3, in the WT series alone, only 0.009 gene was unidentified, and in screens against all three backgrounds together, 0.001. However, it does not necessarily mean that new genes will not be discovered. For instance, the ENU/WT screen identified *drop-6* and *drop-7* which were predicted nonexistent by Poisson analysis of the EMS/WT screen; the EMS/WT screen identified *drop-3*, *drop-5*, and *drop-9* predicted nonexistent by the ENU/WT screen; and the *acs-22* screen identified *drop-4* and *drop-8* predicted nonexistent in the WT screen. *drop-4*, *drop-5*, *drop-7*, *drop-8*, and *drop-9* are of very low allele frequency with just one or two alleles. Interestingly, these alleles of *drop-4*, *drop-5*, *drop-8*, and *drop-9* have a brood size significantly lower than wild type and other *drop* mutants (Figure S4) due to either sterility or embryonic/larval lethality. These alleles may be rare partial loss-of-function mutations. Thus, it seems unlikely that major complementation groups with high allele frequency were missed in our system. Any missing ones may involve sterility and or lethality genes, which may be discovered by screening F2 progeny of cloned F1s and by using genetic balancers.

We categorized the nine *drop* genes into four classes according to their effect on peroxisome matrix protein import, dependence on ACS-22-DGAT-2-mediated LD growth, and occurrence of direct LD fusion. The rate-limiting enzyme complex ACS-22/FATP1-DGAT-2 together with glycerol-3-phosphate O-acyltransferase 4 (GPAT4) synthesizes TAG on the surface of LDs and TAG is directly packaged into existing LDs, leading to an enhancement of LD growth and supersized LD formation in *C. elegans*, *Drosophila*, and mammalian cells (Harris *et al.* 2011; Xu *et al.* 2012; Wilfling *et al.* 2013). Similar to peroxisomal fatty acid β-oxidation mutants *maoc-1*/*dhs-28*/*daf-22*, class I and class II mutants are ACS-22-DGAT-2-dependent and hydrolysis-resistant. Class I and class II thus represent a genetic model of enhanced LD growth and inhibited LD hydrolysis. Class I *drop-5* and *drop-9* likely encode PRX proteins involved in peroxisome import. Class II *drop-2*, *drop-3*, *drop-6*, and *drop-7* may encode peroxisomal fatty acid β-oxidation enzymes not yet identified in *C. elegan*s (Zhang *et al.* 2010a). Class III *drop-1* and *drop-8*, being ACS-22-independent and hydrolysis-sensitive, are apt to represent enhanced LD fusion. LD fusion in *C. elegans* has never been convincingly reported before. Our model of LD fusion is particularly useful because it is controllable by a temperature up-shift and the process is fast, resembling “typical LD fusion” rather than “atypical LD fusion” in yeast, *Drosophila*, and mammalian cells (Fei *et al.* 2011; Gong *et al.* 2011; Krahmer *et al.* 2011). It is interesting that a restrictive temperature of 30° is required for triggering the fusion process even in *drop-1* null alleles (unpublished results), suggesting that LD fusion may be a temperature-sensitive process with unusual thermodynamics. The factors contributing to this unusual thermodynamics may include phospholipid composition, fatty acid chain length, degree of unsaturation, and branching in the mono-layered membrane of LDs (Fei *et al.* 2011; de Mendoza 2014). In natural environments, *C. elegans* frequently encounters extreme temperatures which have been shown to play important roles in several physiological processes including dauer formation, synaptic vesicle endocytosis, and temperature adaptation (Golden and Riddle 1984; Sato *et al.* 2009; Svensk *et al.* 2013; Ma *et al.* 2015). Further studies of our model may yield insights into regulatory mechanisms of LD fusion. Class IV *drop-4*/*sams-1* is ACS-22-independent and hydrolysis-resistant. It was thought that through up-regulating the expression of ACS-22, DGAT-2, fatty acid synthase, desaturases, and elongases, *sams-1* mutation decreases phosphatidylcholine (PC) synthesis and increases TAG synthesis (Li *et al.* 2011; Walker *et al.* 2011; Ehmke *et al.* 2014). But it is still poorly understood why TAG increase results in LD size increase but not number increase. The ACS-22-independence of *drop-4*/*sams-1* indicates that supersized LDs do not form by enhanced LD growth. However, a possibility of ACS-22-independent LD growth cannot be excluded and evidence for enhanced LD fusion is lacking. Detailed molecular analysis of these four classes of genes in LD size regulation and fat metabolism will be published elsewhere.

## Supplementary Material

Supplemental Material
