# Supplementary material for: A Genetic Screen for Mutants with Supersized Lipid Droplets in Caenorhabditis elegans
Source: G3 (Bethesda). 2016 Jun 1;6(8):2407–19. doi: 10.1534/g3.116.030866 (PMC4978895; doi:10.1534/g3.116.030866)
Supplement: Supplemental Material [file supp_6_8_2407__index.html]

A Genetic Screen for Mutants with Supersized Lipid Droplets in Caenorhabditis elegans — A Genetic Screen for Mutants with Supersized Lipid Droplets in Caenorhabditis elegans — Supplemental Material 

# A Genetic Screen for Mutants with Supersized Lipid Droplets in *Caenorhabditis elegans*

## Supplemental Material for Li *et al.*, 2016

**Files in this Data Supplement:**

- Table S1 - Mutation spectra of two different mutagens targeting at *maoc-1/dhs-28/daf-22/prx-10*. (.pdf, 72 KB)
- File S1 - This File contains legends for all Supplemental Figures. (.pdf, 131 KB)
- Figure S1 - GFP::DGAT-2 specifically labels LDs. (.jpg, 1,442 KB)
- Figure S2 - BODIPY labels LDs in addition to LROs. (.jpg, 1,045 KB)
- Figure S3 - SNP mapping of *drop* genes. (.jpg, 1,538)
- Figure S4 - Brood size of wild type and *drop* mutants. (.jpg, 748 KB)
