# Supplementary material for: A Genetic Screen for Mutants with Supersized Lipid Droplets in Caenorhabditis elegans
Source: G3 (Bethesda). 2016 Jun 1;6(8):2407–19. doi: 10.1534/g3.116.030866 (PMC4978895; doi:10.1534/g3.116.030866)
Supplement: Supplemental Material [file supp_g3.116.030866_TableS1.pdf]

Table S1 Mutation spectra of two different mutagens targeting at *maoc-1/dhs-28/daf-22/prx-10*

Mutation / Mutagen	GC→AT	GC→CG	GC→TA	AT→GC	AT→CG	AT→TA	Indel
EMS [a]	93.7		3.1		3.1		
ENU [b]	61.5		7.7	7.7		15.4	7.7
EMS [c]	95	0.4	2	1		2	
ENU [d]	42			42	8	8	N/A
ENU [e]	52			23	16	10	N/A

Values are percentages of mutation sites. Void, not found; N/A, data unavailable.

[a] Data from this study; N=32 of *maoc-1/dhs-28/daf-22/prx-10*

[b] Data from this study; N=13 of *maoc-1/dhs-28/daf-22/prx-10*. ENU concentration, 0.5 mM.

[c] Data from Anderson 1995; N=238.

[d] Data from De Stasio et al. 1997; Indels were not included due to incomplete analysis. N=12 of *unc-93*. ENU concentrations, 0.25, 0.5, 3.0, 5.0, 10, 15, 20, and 25 mM.

[e] Data from De Stasio and Dorman 2001. N=19 of *egl-30/spe-10/spe-37/sup-9*. ENU concentrations unspecified.
