# Supplementary material for: A Genetic Screen for Mutants with Supersized Lipid Droplets in Caenorhabditis elegans
Source: G3 (Bethesda). 2016 Jun 1;6(8):2407–19. doi: 10.1534/g3.116.030866 (PMC4978895; doi:10.1534/g3.116.030866)
Supplement: Supplemental Material [file supp_g3.116.030866_FileS1.pdf]

Supplemental Figure Legends

Figure S1. GFP::DGAT-2 specifically labels LDs. (A) LDs were purified from *hjSi56*, a transgenic line expressing 3xFLAG::GFP::DGAT-2 under a gut-specific *vha-6* promoter. Total membranes (TM) were also purified. Protein was extracted from LD and TM and 10 μg protein of each was loaded onto SDS-PAGE. As a negative control, equal amount of protein of wild type (WT) LD and TM samples were loaded alongside. Proteins were subjected to Western blot with an anti-FLAG antibody. The 3xFLAG::GFP::DGAT-2 protein of expected MW of 71.2 KD (red arrow) was detected in the *hjSi56*-LD sample but not in the *hjSi56*-TM sample or in the WT samples. (B-E) *hjSi56* was imaged by lambda mode confocal microscopy, GFP signal (green) was separated from autofluorescence signal (red) by linear un-mixing. The GFP signal appears as circled globes while autofluorescence appears as filled globes. Autofluorescent globes are not encircled by GFP::DGAT-2 (arrows). (F) In *glo-4(ok623)* mutants, no autofluorescent globes but GFP::DGAT-2-encircled globes exist. (G) In *glo-4(ok623); hjSi56*, 0% autofluorescent globe exist, while in *hjSi56*, 15.6%. For *hjSi56* and *glo-4(ok623); hjSi56*, fluorescent structures were counted in a 32 μm X 32 μm region that covered a randomly selected part the intestine of each sample. The number of counted structures were 407 of three *hjSi56* samples, and 280 of three *glo-4(ok623); hjSi56* samples. *, $P<0.05$, unpaired two-sample t-test.

Figure S2. BODIPY labels LDs in addition to LROs. (A) *hjSi56* animals were vital stained by a red BODIPY and subjected to confocal microscopy. BODIPY strongly labels a population of organelles that are not encircled by GFP::DGAT-2 (red arrows). BODIPY also weakly labels a population of organelles that are encircled by GFP::DGAT-2 (white arrows). (B) In *glo-4(ok623)* mutants, BODIPY only labels organelles encircled by GFP::DGAT-2 (white arrows). (C) Statistics of the data in (A) & (B). In *hjSi56*, only 78.9% of BODIPY-labeled organelles are encircled by GFP::DGAT-2, while in *glo-4(ok623)*, 99.9% are encircled by GFP::DGAT-2. For *hjSi56* and *glo-4(ok623); hjSi56*, BODIPY-labeled organelles were counted in a 32 μm X 32 μm region that covered a randomly selected part the intestine of each sample. The total number of counted BODIPY-positive organelles were 802 of five *hjSi56* samples, and 495 of six *glo-4(ok623); hjSi56* samples. **, $P<0.01$, unpaired two-sample t-test.

Figure S3. SNP mapping of *drop* genes. (A) *drop-1(ssd2)* was first mapped onto Chr II by SNP mapping. In a SNP interval mapping, nine *drop-1(ssd2)* homozygotes were found to have recombined at SNP Y51A1A (extrapolated genetic position, 21), bringing a Hawaiian SNP (red) into the N2 background (blue). Five of the nine recombinants also recombined at W02B8.5 (21.39). On the other end, five animals recombined at K09E4 (22.4), of which two recombined at W01G7.3 (22.38). Thus, the *drop-1(ssd2)* locus is between W02B8.5 (21.39) and W01G7.3 (22.38). (B-G) SNP interval mapping of *drop-2(ssd16)* on Chr IV, *drop-3(ssd36)* on Chr IV, *drop-5(ssd72)* on Chr X, *drop-6(ssd73)* on Chr X, *drop-8(ssd89)* on Chr III, and *drop-9(ssd213)* on Chr II. Mapping data are diagrammed as in (A).

Figure S4. Brood size of wild type and *drop* mutants. Compared to wild type, *drop-2(ssd14), drop-4(ok2946), drop-5(ssd72)*^{ts}, *drop-7(ssd75), drop-8(ssd89)*^{ts}, and *drop-9(ssd213)* are lower in brood size. Experiments were conducted at 20°, a restrictive temperature for *drop-5(ssd72)*^{ts} and *drop-8(ssd89)*^{ts} in brood size phenotype. **， $P<0.01$; ***, $P<0.001$; unpaired two-sample t-test.
