# Supplementary figures and images for: A Genetic Screen for Mutants with Supersized Lipid Droplets in Caenorhabditis elegans
Source: G3 (Bethesda). 2016 Jun 1;6(8):2407–19. doi: 10.1534/g3.116.030866 (PMC4978895; doi:10.1534/g3.116.030866)

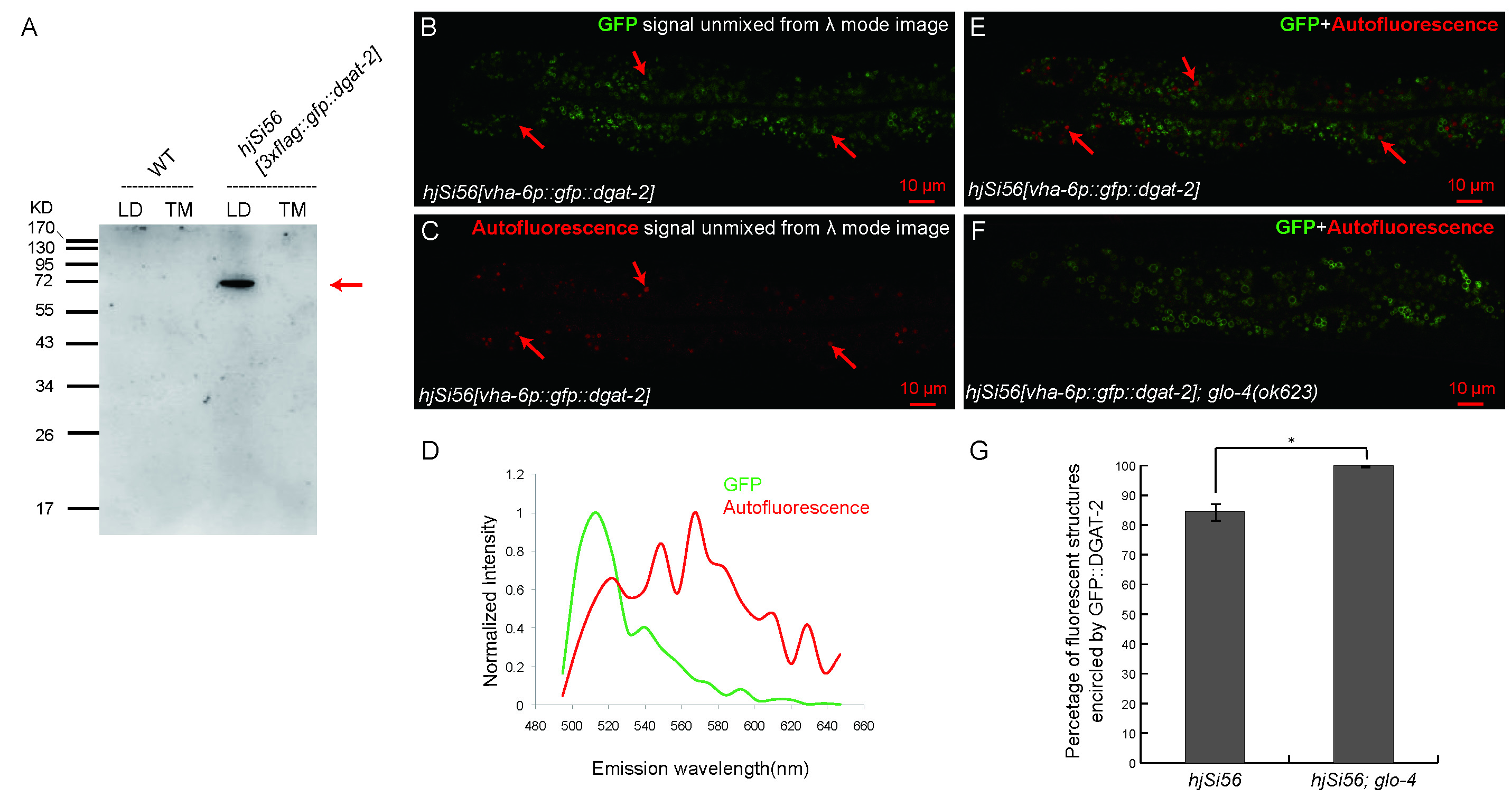

Supplement: Supplemental Material [file supp_g3.116.030866_FigureS1.jpg]

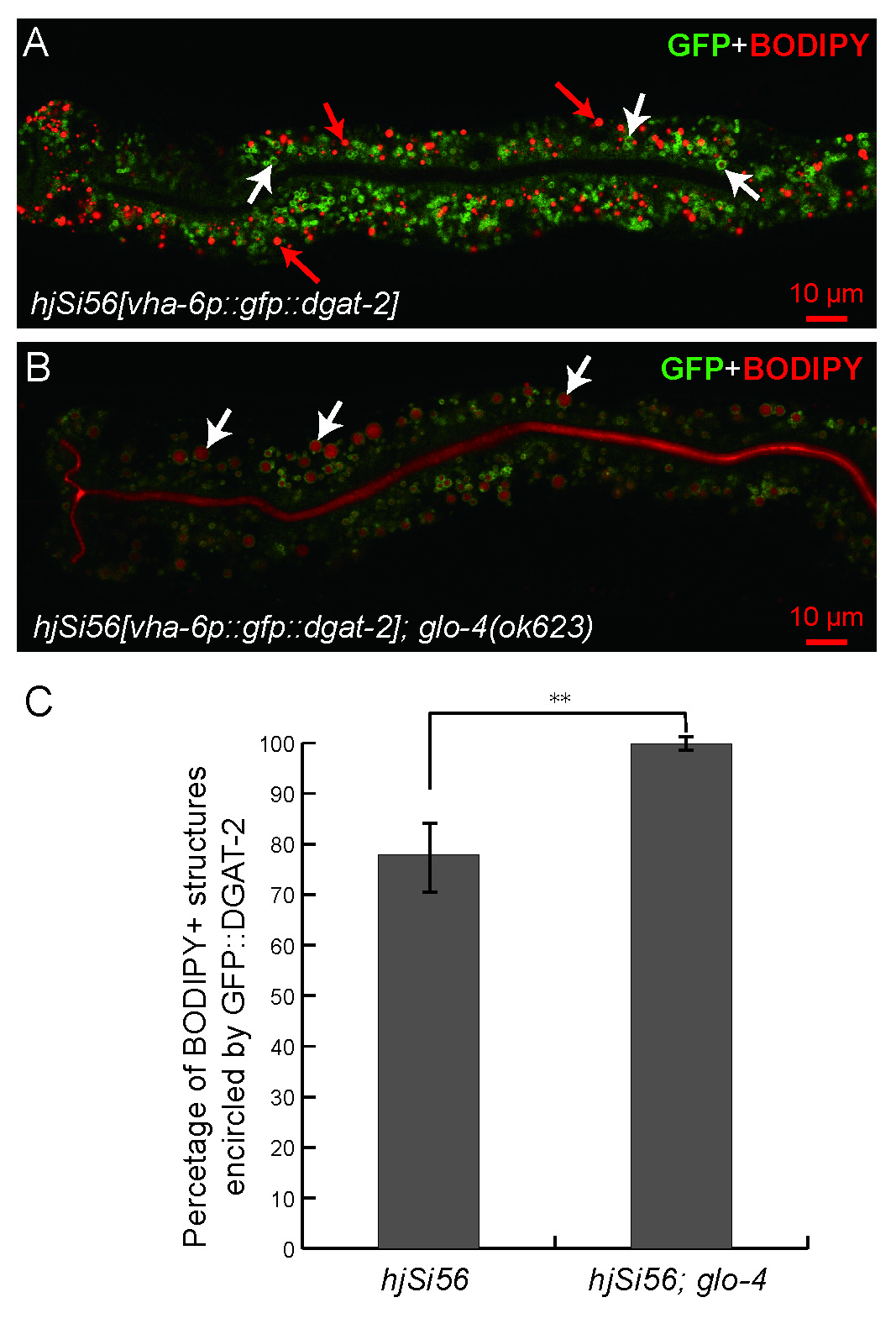

Supplement: Supplemental Material [file supp_g3.116.030866_FigureS2.jpg]

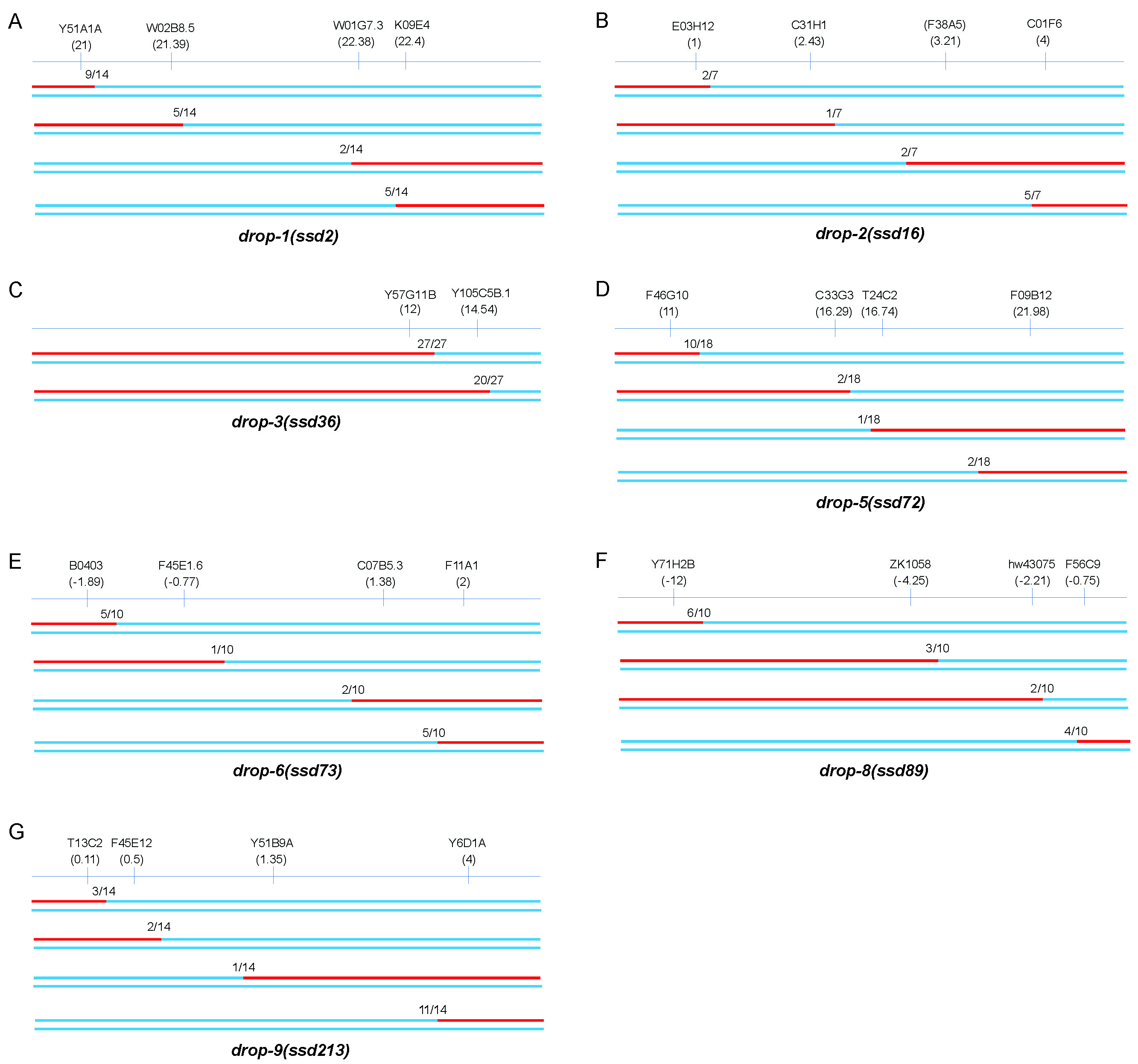

Supplement: Supplemental Material [file supp_g3.116.030866_FigureS3.jpg]

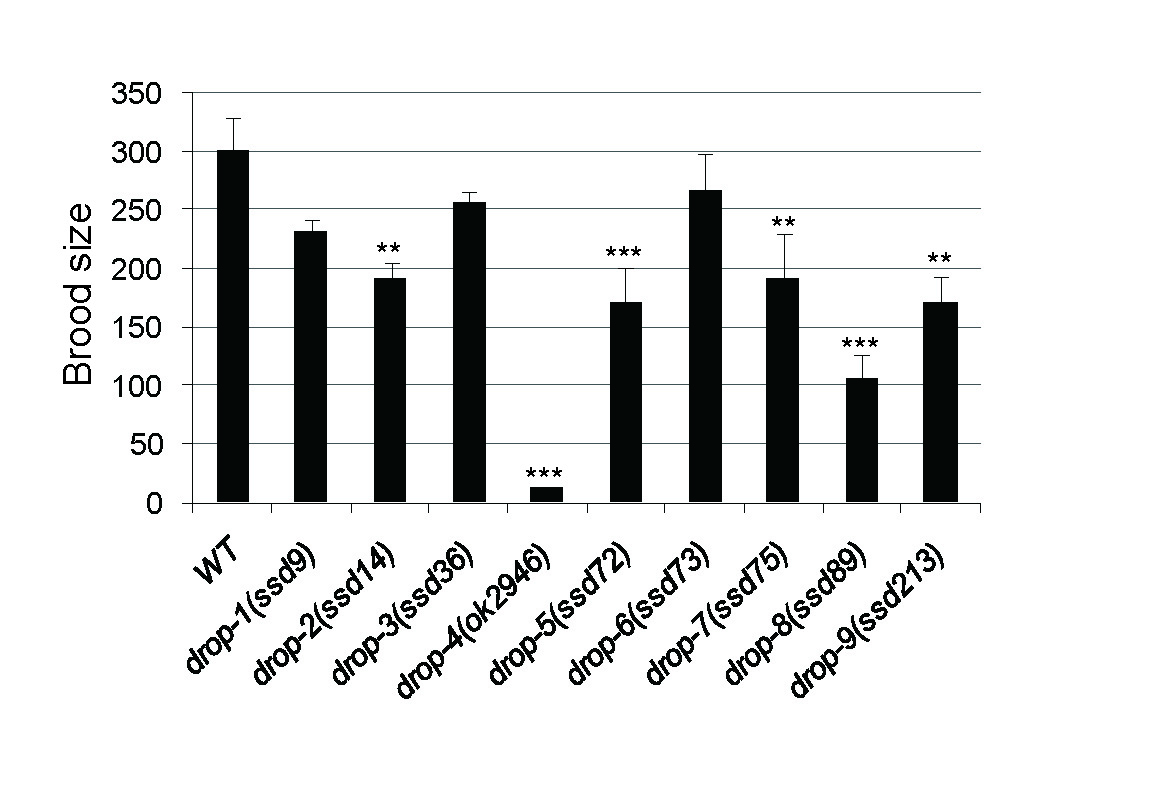

Supplement: Supplemental Material [file supp_g3.116.030866_FigureS4.jpg]
